# Characterization of Microbiota in Endometrial Fluid and Vaginal Secretions in Infertile Women with Repeated Implantation Failure

**DOI:** 10.1155/2019/4893437

**Published:** 2019-05-21

**Authors:** Kotaro Kitaya, Yoko Nagai, Wataru Arai, Yoshiyuki Sakuraba, Tomomoto Ishikawa

**Affiliations:** ^1^Reproduction Clinic Osaka, Grand Front Osaka Tower A 15F, 4-20 Oofuka-cho, Kita-ku, Osaka, 530-0011, Japan; ^2^Varinos Inc., Dai 2 Gotanda Fujikoshi Bldg. 6F, 5-23-1 Higashigotanda, Shinagawa-ku, Tokyo, 141-0022, Japan; ^3^Reproduction Clinic Tokyo, Shiodome City Center 3F, 1-5-2 Higashi-Shinbashi, Minato-ku, Tokyo, 105-7103, Japan

## Abstract

Studies suggest that persisting intrauterine bacterial infectious conditions such as chronic endometritis potentially impair the embryo implantation process. The microbial environment in the female reproductive tract, however, remains largely undetermined in infertile patients with a history of repeated implantation failure (RIF). Using next-generation sequencing, we aimed to characterize the microbiota in the endometrial fluid (EF) and vaginal secretions (VS) in women with RIF. Twenty-eight infertile women with a history of RIF and eighteen infertile women undergoing the first *in vitro* fertilization-embryo transfer attempt (the control group) were enrolled in the study. On days 6-8 in the luteal phase of the natural, oocyte-pickup, or hormone replacement cycle, the paired EF and VS samples were obtained separately. Extracted genomic DNA was pyrosequenced for the V4 region of 16S ribosomal RNA using a next-generation sequencer. The EF microbiota had higher *α*-diversity and broader bacterial species than the VS microbiota both in the RIF and control groups. The analysis of the UniFrac distance matrices between EF and VS also revealed significantly different clustering. Additionally, the EF microbiota, but not the VS microbiota, showed significant variation in community composition between the RIF group and the control group. *Burkholderia* species were not detected in the EF microbiota of any samples in the control group but were detectable in a quarter of the RIF group. To our best knowledge, this is the first study investigating the microbiota in the paired EF and VS samples in infertile women with RIF.

## 1. Introduction

The Human Microbiome Project revealed that bacterial cells account for ~3% of total human body weight and are at an equal level in number to human somatic cells. While the bacterial communities in the human body contribute to health, their imbalance predisposes to a wide variety of diseases [1].

*Lactobacillus* species are classically known to dominate the vaginal cavity in premenopausal women [2]. Vaginal *Lactobacilli* play a role in the maintenance and homeostasis of the local microbial milieu by dropping pH through production of lactic acid. Meanwhile, the human uterine cavity has been long believed to be germfree. Recent studies, however, proved the presence of a microbiota in the uterine cavity, which is also characterized by *Lactobacillus*-dominant composition [3–5]. Moreover, it was demonstrated that the status of the *Lactobacillus*-dominant (90% or more) microbiota in the endometrial fluid (EF) was favorable for embryo implantation in the subsequent *in vitro* fertilization-embryo transfer (IVF-ET) treatment in infertile women. On the contrary, non-*Lactobacillus*-dominant microbiota is associated with a poor reproductive outcome including implantation failure and miscarriage [6], supporting the idea that endometrial microbial composition is a key determinant for a successful embryo implantation process.

Repeated implantation failure (RIF) is an infertile condition recognized as serial failed conception following three or more transfer cycles with good-quality embryos [7]. RIF occurs in 15-20% of infertile couples undergoing an IVF-ET program [8]. RIF potentially originates in aberrant embryonic factors (such as chromosomal abnormalities, mitochondrial DNA quantity, and oxidative stress) [9–12], impaired endometrial receptivity (such as hydrosalpinx, endometrial polyps, distorted uterine cavity, and chronic endometritis (CE)) [13–16], and systemic factors (such as thrombophilic and immunological factors) [17, 18]. Despite the accumulating evidence that *Lactobacillus* species are essential for the integrity of both the vaginal and the uterine cavity environments, the relationship between the vaginal secretions (VS) microbiota and the EF counterpart within the same infertile individuals remains largely unknown. Using next-generation sequencing, we aimed to compare the diversity of the microbiota in the paired EF and VS samples and characterize their dysbiosis in patients with a history of RIF.

## 2. Materials and Methods

### 2.1. Subjects

This was a preliminary analysis of an ongoing case-control study, which was approved by the Ethical Committee of the Institutional Review Board (Approval Number 2017-02) and registered on the University Hospital Medical Information Network-Clinical Trial Registration, Japan (UMIN000029449) on the 6th of October 2017. Under a given written informed consent, infertile patients with a history of RIF (RIF group, *n* = 28) and those undergoing the first IVF attempt (control group, *n* = 18) in high-volume centers (>2000 oocyte-pickup cycles per year) were enrolled into the study. They had undergone infertility examinations including hysterosalpingogram, hysteroscopy, thyroid functions, and thrombophilic and immunological factors. According to Veeck's classification [19], morphologically good cleavage-stage embryos were defined as day 3, grade 1 or 2, seven-to-nine-cell embryos. According to Gardner's score [20], morphologically good blastocysts were defined as day 5 blastocysts with a score of 3BB or above. Serum human chorionic gonadotropin (hCG, Tosoh Co., Shunan, Japan) was measured on the eleventh day after transfer of day 3 embryos or on the ninth day after transfer of day 5 blastocysts. According to the manufacturer's instruction, a value less than 0.5 IU/L was regarded as a negative pregnancy test. RIF was defined as serial negative pregnancy tests following transfer of five or more morphologically good cleavage-stage embryos and/or blastocysts.

### 2.2. Sample Collection

Endometrial biopsy samples were obtained in the proliferative phase (on days 6-12) of the menstrual cycle using a 3 mm width curette (Atom Medical, Tokyo, Japan). On days 6-8 after luteinizing hormone surge in the natural cycle, or hCG trigger in the oocyte-pickup cycle, or on day 5 following initiation of luteal support in the hormone replacement cycle, the paired EF and VS samples were obtained carefully avoiding contamination. In brief, the perineum was cleansed twice using sterilized cotton balls soaked in benzalkonium chloride solution. A bivalve speculum was inserted slowly into the vaginal cavity to visualize the uterine cervix sufficiently. The VS samples were obtained from the vaginal mucosa from all directions using an OMNIgene accessory swab (DNA Genotek Inc., Ottawa, ON, Canada) and solubilized into a collection tube containing stabilizing liquid (DNA Genotek Inc.). After removing the mucous, the vaginal cavity and cervix were cleaned twice using sterilized cotton balls soaked in benzalkonium chloride solution. A MedGyn Pipette IV (MedGyn Products Inc., Addison, IL, USA) was used for EF sample collection. Avoiding contact between the speculator and vaginal wall, a pipette was inserted slowly from the cervical os into the uterine cavity until it reached the fundus uteri. The EF samples were then carefully aspirated and soaked into another collection tube.

### 2.3. Histopathologic/Immunohistochemical Examinations for CE

Endometrial biopsy samples were fixed overnight in 4% paraformaldehyde (in phosphate buffer, pH 7.3) and embedded in paraffin. The sections (4 *μ*m thickness) on slide glasses were dewaxed in limonene (Falma Inc., Tokyo, Japan), rehydrated in a graded series of ethanol (in phosphate-buffered saline, pH 7.4), and subjected to microwave pretreatment in citrate buffer solution (pH 6.0) for 5 minutes for antigen retrieval and immersion in 3% hydrogen peroxide for 5 minutes for endogenous peroxidase activity blocking. After being washed, the sections were soaked in 10% fetal calf serum (SAFC Biosciences, Lenexa, KS, USA) for 10 minutes to minimize nonspecific antibody binding and incubated with the ready-to-use mouse monoclonal IgG antibody against human CD138 (a plasmacyte marker, B-A38; Nichirei, Tokyo, Japan) or control mouse IgG. After being washed three times, the immunoreactivity was developed using a LSAB kit (Dako, Kyoto, Japan). Following hematoxylin counterstaining, the sections were observed by an experienced gynecologic pathologist under a light microscope (400x magnification). Stromal CD138+ cells with a nucleic heterochromatin pattern were enumerated in 20 or more high-power fields. The endometrial stromal plasmacyte density index was calculated as the sum of the stromal CD138+ cell counts divided by the number of the high-power fields evaluated. CE was diagnosed as 0.25 or more ESPDI, as previously described [21].

### 2.4. DNA Extraction and Sequencing

Both the EF and VS samples were treated with proteinase K (Beckman Coulter Inc., Brea, CA, USA) containing 100 mg/mL lysozyme solution (Sigma-Aldrich, Darmstadt, Germany) and 100 mg/mL RNase A (Sigma-Aldrich). The genomic DNA was extracted using an Agencourt Genfind v2 Blood & Serum DNA Isolation Kit (Beckman Coulter Inc.). The double-stranded DNA concentration was quantified fluorometrically with a Qubit dsDNA HS Assay Kit (Thermo Fisher Scientific Inc., Waltham, MA, USA). The variable region 4 (V4) hypervariable region of the bacterial 16S rRNA gene was amplified from the specimen DNA by using a modified primer pair, 515f (5′-TCGTCGGCAGCGTCAGATGTGTATAAGAGACAGGTGYCAGCMGCCGCGGTAA-3′) and 806rB (5′-GTCTCGTGGGCTCGGAGATGTGTATAAGAGACAG-GGACTACNVGGGTWTCTAAT-3′), with Illumina Nextera XT (Illumina Inc., San Diego, CA, USA) adapter overhang sequences [22]. Polymerase chain reaction (PCR) was performed with 25 ng DNA, 200 *μ*mol/L 4-deoxynucleotide triphosphates, 400 nmol/L of each primer, 2.5 U of FastStart HiFi polymerase, 4% of 20 mg/mL BSA, 0.5 mol/L betaine, and the appropriate buffer with MgCl_2_ supplied by the manufacturer (Sigma-Aldrich). Thermal cycling consisted of initial denaturation at 94°C for 2 minutes followed by 30 cycles of denaturation at 94°C for 20 seconds, annealing at 50°C for 30 seconds, extension at 72°C for 1 minute, and final extension at 72°C for 5 minutes. The amplicon mixture was purified using Agencourt AMPure XP (Beckman Coulter Inc.). Purified PCR samples were multiplexed using a dual-index approach with the Nextera XT Index Kit v2 according to the Illumina 16S Metagenomic Sequencing Library Preparation protocol. The indexing PCR was performed with a KAPA HiFi HotStart ReadyMix (Kapa Biosystems, Boston, MA, USA) in a 50 *μ*L reaction volume, and purification was then performed with Agencourt AMPure XP beads. The final library was paired-end sequenced at 2 × 200 bp using a MiSeq Reagent Kit v3 on the Illumina MiSeq platform. The ZymoBIOMICS Microbial Community Standard (Zymo Research, Orange, CA, USA) containing a mixture of *Pseudomonas*, *Escherichia*, *Salmonella*, *Lactobacillus*, *Enterococcus*, *Listeria*, *Bacillus*, and two yeast species *Saccharomyces* and *Cryptococcus* was used as a positive control. UltraPure™ DNase/RNase-Free Distilled Water (Thermo Fisher Scientific Inc.) was used as a blank control.

Using EA-Utils fastq-join [23], a median 291-base pair merged sequence length was obtained. The quality control of the merged sequence was performed using USEARCH v10.0.240 [24] to remove PhiX reads, truncate primer-binding sequences, and discard sequences with <100 bp length and sequence quality < Q20. Quantitative Insights Into Microbial Ecology (QIIME) 1.9.1 [25] was used with default parameters for quality filtering, chimera check, clustering sequences into operational taxonomic units (OTUs), and assignment of taxonomy. The sequences were clustered into OTUs by open-reference OTU picking strategy using the UCLUST method based on 97% sequence identity. Taxonomy was assigned to each OTU using the Ribosomal Database Project Classifier [26] with a 0.50 confidence threshold against the Greengenes database version 13_8 [27]. The following 15 bacterial taxa (*Acidovorax*, *Acinetobacter*, *Chryseobacterium*, *Citrobacter*, *Elizabethkingia*, *Escherichia*, *Flavobacterium*, *Janthinobacterium*, *Leptothrix*, *Methylobacterium*, *Pseudomonas*, *Rhodococcus*, *Sphingomonas*, *Stenotrophomonas*, and *Yersinia*), which are known as contaminants found in a blank control [28–30], were excluded from ES samples using QIIME.

### 2.5. Statistics

*α*-Diversity including the Shannon index, Chao1 richness, and observed species were calculated at the 1,000-th sequence in QIIME. The unweighted and weighted UniFrac distances were used to inspect the phylogenetic-based *β*-diversity and principal coordinate analysis plot based on rarified sequences for 1,000 [31]. The plots of *α*- and *β*-diversity were generated in QIIME, and *β*-diversity between the groups was compared using the permutational multivariate analysis of variance (PERMANOVA) test. Pearson's correlation analysis was applied for comparison between the EF and the VS microbiota within the same individual. Fisher's exact test was conducted to compare taxon-relative abundances between the control and the RIF group. A *p* value less than 0.05 was regarded as statistically significant.

## 3. Results

### 3.1. Characteristics of Infertile Patients

The demographics of the infertile patients enrolled were summarized in Table 1. All the patients were from the Japanese population. There were no cigarette smokers and obese women (body mass index > 30) in both groups. CE was detected in 6 of 28 (21.4%) patients in the RIF group and 2 of 18 (11.1%) patients in the control group. The prevalence of CE was higher in the RIF group than in the control group but did not reach a significant level (*p* = 0.38, relative risk 1.93, 95% CI 0.43 to 8.53). The association between CE and specific EF/VS microbiota was not found in this small cohort setting.

### 3.2. Sequencing Result of EF and VS Samples

The paired EF and VS samples were obtained from the RIF group (*n* = 28; 17 in the natural cycle, 4 in the oocyte-pickup cycle, and 7 in the hormone replacement cycle) and the control group (*n* = 18; 8 in the natural cycle, 4 in the oocyte-pickup cycle, and 6 in the hormone replacement cycle) and were subjected to sequencing. A total of 12,570,533 sequence reads were obtained with a mean 106,308 reads per sample (range, 5,969–297,391) in EF and 166,965 reads per sample (range, 60,484–535,057) in VS. The microbiota obtained in EF was a mean 26,725 OTU-assigned sequences per sample (range, 1,065–43,657), whereas the microbiota obtained in VS was a mean 37,712 OTU-assigned sequences (range, 18,232–43,936).

### 3.3. Comparison of EF versus VS Microbiota in Infertile Patients

**Figure 1:**
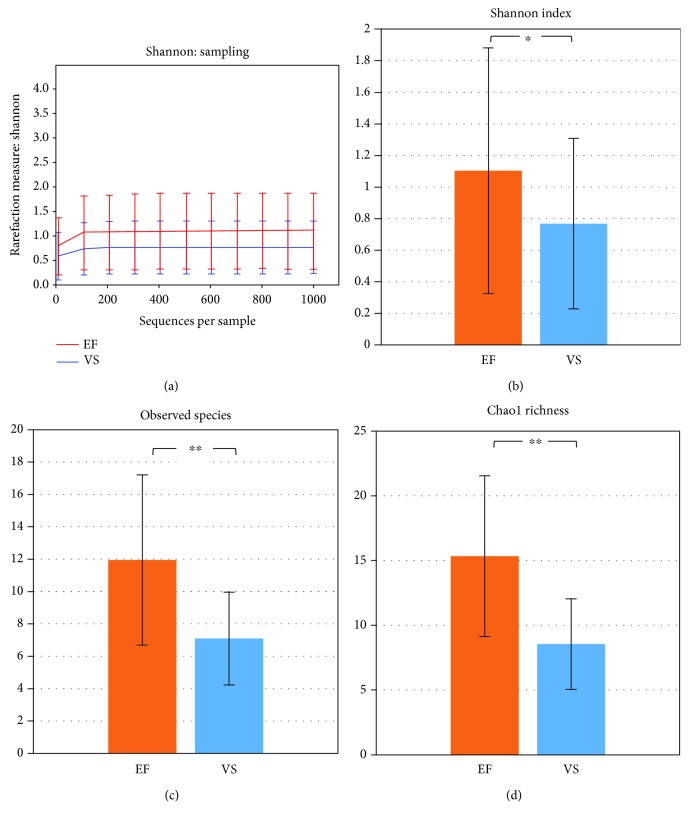
Calculation of *α*-diversity values for comparison of bacterial communities between EF and VS. (a) Rarefaction analysis of sequences per sample in EF and VS. Comparison of Shannon index (b), mean number of observed species (c), and Chao1 richness (d) between EF and VS. Each graph represents mean (column) and SE (bars). ^∗^*p* < 0.05 and ^∗∗^*p* < 0.01 by two-tailed *t-*test.

**Figure 2:**
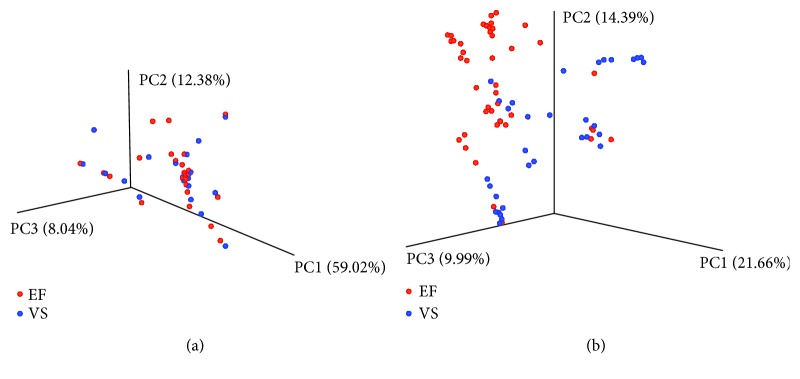
Principal coordinate analysis plotting of EF and VS microbiota in whole samples (*n* = 46). The plots were generated using weighted (a) and unweighted (b) UniFrac distance metrics.

Both the EF and the VS microbiota were highly correlated within the same infertile individual (average Pearson correlation coefficient for all subjects, *r* = 0.952). *α*-Rarefaction analysis demonstrated that the Shannon index was highly stable above 100 sequences, indicating that enough sequencing was conducted to analyze the diversity of both the EF and the VS microbiota (Figure 1). Assessment of the Shannon index revealed that the EF microbiota (mean ± SE, 1.104 ± 0.777) was more diverse (*p* = 0.020) than the VS microbiota (mean ± SE, 0.768 ± 0.540) at 1,000 reads in infertile patients. In addition, the number of the bacterial species observed in the EF microbiota (mean ± SE, 11.950 ± 5.262) was significantly higher (*p* < 0.0001) compared to that in the VS microbiota (mean ± SE, 7.091 ± 2.865) in both groups (Figure 1). Richness of bacterial community measured by Chao1 richness was higher (*p* < 0.001) in the EF microbiota (15.330 ± 6.214) compared with the VS microbiota (8.550 ± 3.494). Analysis of variance to partition UniFrac distance matrices between EF and VS revealed significantly different clustering (*p* = 0.001) (Figure 2).

### 3.4. Comparison of EF and VS Microbiota between the RIF Group and the Control Group

**Figure 3:**
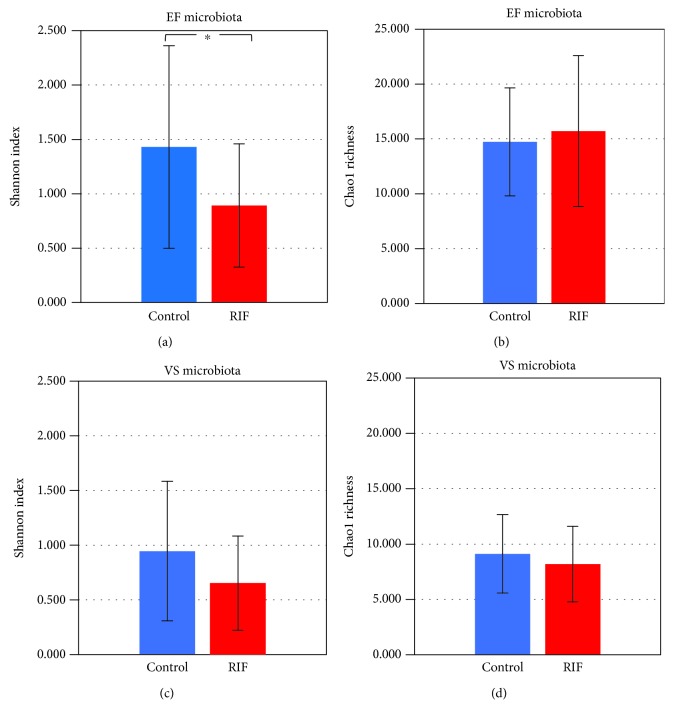
Comparison of *α*-diversity values in the control (*n* = 18) and RIF (*n* = 28) groups. Shannon index (a, c) and Chao1 richness (b, d) of EF (a, b) and VS (c, d) microbiota. Each graph represents mean (column) and SE (bars). ^∗^*p* < 0.05 by two-tailed *t-*test.

**Figure 4:**
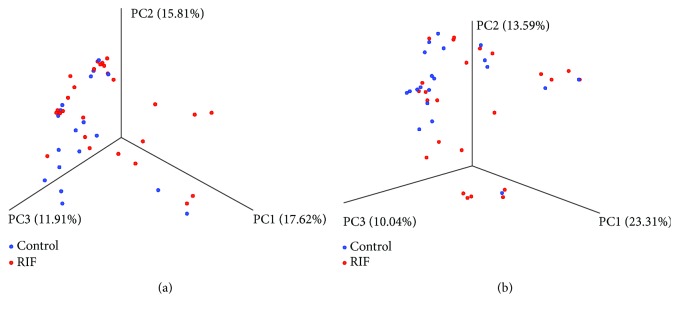
Principal coordinate analysis plotting of unweighted UniFrac distance metrics comparing EF (a) and VS (b) microbiota between the control (*n* = 18) and RIF (*n* = 28) groups.

The Shannon index of the EF microbiota in the RIF group (mean ± SE, 0.893 ± 0.567) was significantly lower (*p* = 0.02) than that in the control group (mean ± SE, 1.431 ± 0.931) (Figure 3). The Shannon index of the VS microbiota in the RIF group (mean ± SE, 0.654 ± 0.431) was comparable (*p* = 0.07) to that in the control group (mean ± SE, 0.946 ± 0.637). The unweighted UniFrac distance of the bacterial community in the EF microbiota showed a significant difference between the RIF group and the control group (*p* = 0.0089). Meanwhile, the unweighted UniFrac distance of the bacterial community in the VS microbiota was similar between the two groups (*p* = 0.38) (Figure 4).

### 3.5. Comparison of Bacterial Species in EF and VS Microbiota between the RIF Group and the Control Group

**Figure 5:**
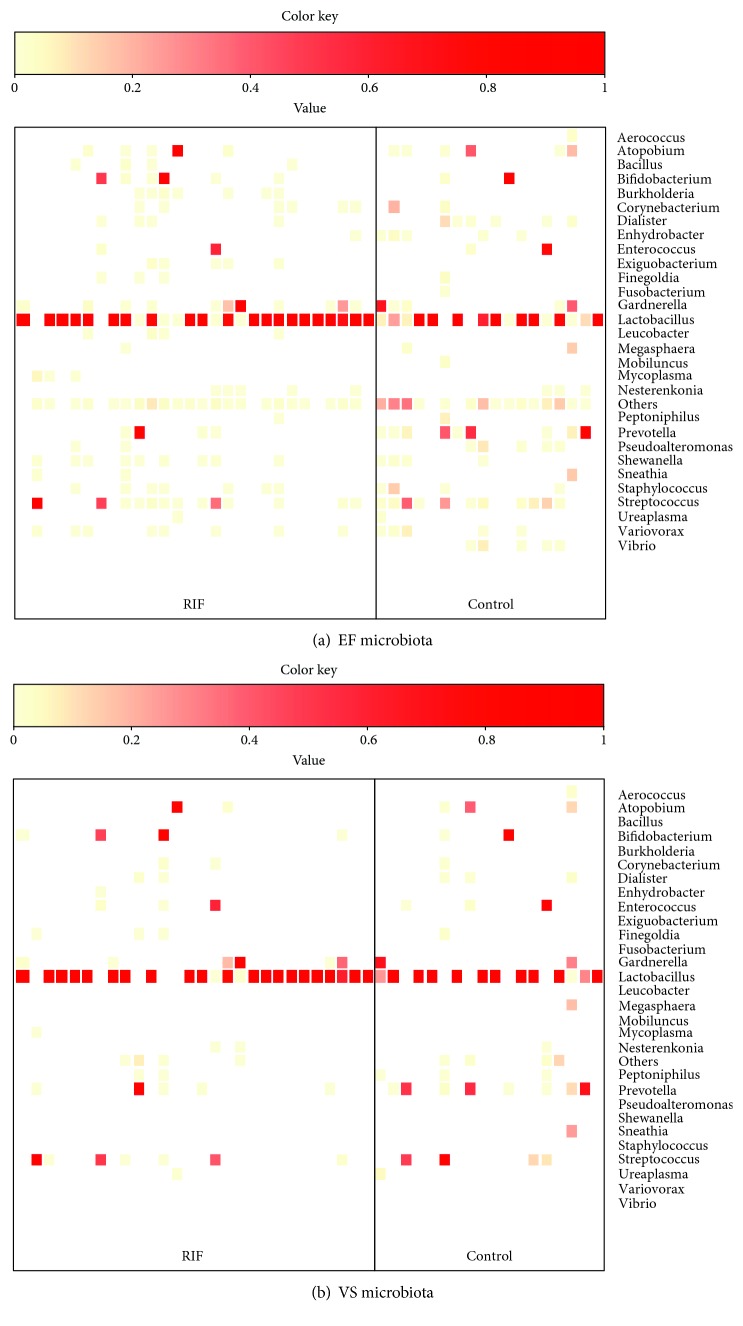
Heatmap representing dominant bacterial genera found in the EF (a) and VS (b) in the RIF and control groups. The rows show bacterial genera in alphabetical order, and the columns represent subjects. For each subject, the dominant genera are shown in red. Bacteria with a total rate less than 0.1% are not shown.

*Lactobacillus*-dominated EF microbiota, defined by >90% *Lactobacillus* genus status, was observed at a higher rate in the RIF group (64.3%, 18/28) than in the control group (38.9%, 7/18), although the difference did not reach a significant level (*p* = 0.13, odds ratio 2.83, 95% CI 0.83-9.61) (Figure 5). Similar results were obtained from the VS microbiota where 67.9% (19/28) in the RIF group and 44.4% (8/18) in the control group represented *Lactobacillus*-dominated microbiota (*p* = 0.14, odds ratio 2.64, 95% CI 0.78-8.96). The detection rate of *Gardnerella* in the EF microbiota was 39.3% (11/28) in the RIF group and 27.7% (5/18) in the control group (*p* = 0.53, odds ratio 1.68, 95% CI 0.47-6.05). *Burkholderia* was not detected in any of the EF microbiota in the control group (0/18) but was detectable in 25% (7/28) of the RIF group (*p* = 0.032, odds ratio 12.91). There were no significant differences in the detection rate of the specific bacterial species in the VS microbiota between the two groups.

## 4. Discussion

Several investigators evaluated the bacterial communities in the endometrium and vagina using microbiota in infertile women with various causes [4, 6, 32–37]. To our best knowledge, this is the first study investigating the microbiota in the paired EF and VS samples in infertile patients with a history of RIF.

We demonstrate that the bacterial species in EF and VS are similar within the same individual. However, the diversity measurements such as the Shannon index, observed species, and Chao1 richness indicate that EF has a higher *α*-diversity than VS. This finding was supported by the analysis of the UniFrac distance, which demonstrated that the bacterial communities were fairly different between EF and VS. Although the possible impact of the endometrial biopsy procedure on the subsequent EF/VS sample status cannot be fully denied, we found reassuring results that there was no endometrial thinning or hemorrhage on the day of the EF/VS aspiration.

Interestingly, *Burkholderia* was not detectable in infertile women undergoing the first IVF-ET attempt but in a quarter of those with a history of RIF. *Burkholderia* is a genus of Proteobacteria, of which members include *Burkholderia pseudomallei*, a microorganism responsible for melioidosis [38], and *Burkholderia cepacia*, a pathogen causing serious pulmonary infections in patients with cystic fibrosis [39]. *Burkholderia* is usually resistant to multiple antibiotics [40]. The literature on this bacterium in the human female reproductive tract is scant. While some studies demonstrated that *Burkholderia* species are the common environmental contaminants which are frequently detectable in the uterine cavity of levonorgestrel intrauterine contraceptive system users [41], a case report suggests that *Burkholderia* may be one of the potential pathogens causing tuboovarian abscess [42]. The impact of *Burkholderia* on endometrial receptivity awaits further study.

The human endometrium is regulated throughout the menstrual cycle under the influence of ovarian steroids. Previous reports found that the endometrial microbiota profiles were stable across the menstrual cycle, between the menstrual cycles, and during the shift from the prereceptive phase (LH+2) to the receptive phase (LH+7) in most women. Some fluctuation, however, was seen in the acquisition of the endometrial receptivity in a fraction (4 of 22) of the subjects [6, 33]. The strength of our study is that we fixed the endometrial sampling period to the window of implantation (on days 6-8 after natural luteinizing hormone surge or hCG trigger or on day 5 following initiation of luteal support in the hormone replacement cycle). While the proportion of the pathogens was at a similar level between the paired EF and VS samples, there was a marked variance between the individuals. One potential confounding factor for this variance is inclusion of three different types of the cycles (natural, hCG-triggered, and hormone replacement cycles).

The limitation of this research is that the study design is cross-sectional. Given that the control group (infertile patients undergoing the first IVF-ET attempt) may include some prospective RIF cohort, longitudinal investigations are required. A potential bias is the contamination of the endocervical secretions and VS in the process of EF sampling [3, 4], although the EF microbiota was not suspected to be brought from the VS microbiota as some differences in the bacterial community were noted between the EF and VS samples within the same individuals. An association between CE and EF/VS microbiota is anticipated in infertile women with RIF, but we were unable to find it in this small sample size. Larger studies are required to evaluate the relationship between the female reproductive tract microbiota and CE.

## 5. Conclusions

To the best of our knowledge, this is the first study investigating the microbiota in the paired EF and VS samples in infertile women with a history of RIF, along with those undergoing the first IVF-ET attempt. This work will facilitate the understanding of the microbial etiology in the female reproductive tract of the infertile patients with RIF.

## Figures and Tables

**Table 1:** Demographics of infertile patients with the RIF and control groups.

	RIF group (*n* = 28)	Control group (*n* = 18)
Age (years) (mean ± SD)	38.7 ± 3.2	37.6 ± 4.2
Body mass index (kg/m^2^) (mean ± SD)	21.8 ± 1.7	22.4 ± 2.1
Gravidity (median (range))	0 (0-4)	0 (0-3)
Parity (median (range))	0 (0-1)	0 (0-1)

Infertility diagnosis^a^		
Male factor	8 (28.6%)	6 (33.3%)
Polycystic ovarian syndrome	9 (32.1%)	4 (22.2%)
Endometriosis	5 (17.9%)	4 (22.2%)
Tubal factor	4 (14.3%)	5 (27.8%)
Unexplained	9 (32.1%)	3 (16.7 %)
Diminished ovarian reserve	1 (3.6%)	0 (0%)

Controlled ovarian stimulation protocol^b^		
Short GnRH agonist cycle	31 (76.1%)	—
Long GnRH agonist cycle	5 (0.7%)	—
Ultralong GnRH agonist cycle	1 (0%)	—
Flexible GnRH antagonist cycle	41 (27.5%)	—
Mild stimulation cycle	8 (0.7%)	—
Natural cycle	2 (0%)	—

Past embryo transfer history (mean ± SD)		
Number of cycles	5.5 ± 0.4	—
Number of embryos transferred	8.1 ± 0.6	—
Number of morphologically good embryos transferred	5.6 ± 0.6	—
Number of assisted hatching use	5.7 ± 0.8	—
Number of hyaluronan-rich medium use	3.4 ± 0.5	—

Footnotes: ^a^Totals are not 100 percent due to some patients having more than one diagnosis. ^b^Totals are not 100 percent due to some patients undergoing more than one controlled ovarian stimulation/oocyte-pickup cycle. Abbreviations: RIF: repeated implantation failure; SD: standard deviation; GnRH: gonadotropin-releasing hormone.

## Data Availability

The data used to support the findings of this study are included within the article.
